# Account‐Holding Intensity in the EU Accountability Landscape: A Comprehensive Review of EU agencies' Institutional Accountability Relationships*

**DOI:** 10.1111/jcms.13367

**Published:** 2022-06-20

**Authors:** Benjamin Leidorf‐Tidå, Thijs de Boer

**Affiliations:** ^1^ Department of Political Science and Public Administration Vrije Universiteit Amsterdam Amsterdam the Netherlands

**Keywords:** accountability, account‐holding intensity, EU, EU agencies, mixed‐methods

## Abstract

In this article, we propose a novel conceptualization of account‐holding intensity – defined as both the frequency and diligence of account‐holding – as an instrument for analysing the behaviour of account‐holders in the accountability landscape of EU agencies. We examine the account‐holding intensity of six major institutional EU account‐holders through a complementary mixed‐methods approach that combines quantitative survey data and qualitative interview data collected from directors and senior managers of EU agencies. Account‐holding intensity is measured through the survey data, with the interview data providing detailed insight into why some account‐holders are more/less active and/or diligent than others. The survey and interview data are furthermore triangulated with in‐depth interviews with account‐holders and unobtrusive indicators of account‐holding intensity. This amounts to a comprehensive empirical review of the EU agencies' institutional accountability relationships, which reveals how different account‐holders are driven by different institutional logics that are associated with different account‐holding intensities.

## Introduction

In an EU accountability landscape that is becoming increasingly ‘dense’ (Wille, 2016), EU agencies are subject to scrutiny from many different account‐holders. Among those account‐holders are multiple principals: the European Commission (EC), the European Parliament (EP), and the Council of the European Union (Council) (Dehousse, 2008). In addition, EU agencies are governed by Management Boards (MBs) (Busuioc, 2012), as well as subject to scrutiny from the EU's ‘watchdog institutions’: the European Court of Auditors (ECA) and the European Ombudsman (EO) (Wille and Bovens, 2020).

Prior research on the accountability of EU agencies indicates that the different EU account‐holders execute their account‐holding tasks vis‐à‐vis EU agencies with different intensities. Some studies portray EU account‐holders as active and diligent. The EC has for example been found to have close contact with EU agencies (Egeberg and Trondal, 2017), and the ECA and the EO have both been described as diligent account‐holders (Busuioc, 2013). Other studies portray EU account‐holders as passive in their account‐holding roles. For example, the EP's ‘patchy and unfocused’ (Kluger Dionigi, 2020, p. 74) account‐holding has been found to centre around only a few agencies (Font and Perez Duran, 2016), and some members of MBs have been described as poorly prepared and as lacking interest in account‐holding tasks (Busuioc, 2013).

This article consolidates and challenges knowledge from existing research on the accountability of EU agencies by closely examining the intensity of EU agencies' accountability relationships with six major institutional EU account‐holders (the EC, the EP, the Council, the MBs, the ECA and the EO). Doing so, we aim to answer the following research questions: *To what extent are EU account‐holders active and diligent? What explains the varying intensities with which the different account‐holders execute account‐holding activities?*

In order to address these research questions, we apply a *complementary* mixed‐methods approach (Greene *et al*., 1989). To address the first research question, we use quantitative survey data collected from 75 directors and senior managers (34.1 per cent response rate) of 39 EU agencies (86.7 per cent) to systematically measure and map the account‐holding intensities that EU agencies are subject to from the six account‐holders at the focus of this study. This survey data is complemented with qualitative data from 15 in‐depth interviews, predominantly with executive directors of EU agencies. By contextualizing the survey data and providing detailed insight into why certain account‐holders are (not) active and/or diligent, the interview data allows us to also explore our second research question. To improve the validity of our findings, the survey and interview data is furthermore triangulated with data from 11 in‐depth interviews with account‐holders, as well as with unobtrusive account‐holding indicators: EP written questions (eighth parliamentary term, 2014–19), Council meeting minutes (2015–19), ECA special reports (2015–19), and EO cases opened (2015–19).

Examining questions about account‐holding intensity is of particular importance as pertains to EU agencies. With EU agencies being (semi‐)independent non‐majoritarian institutions (Thatcher and Sweet, 2002), EU agencification has been argued to have propelled a ‘rise of the unelected’ (Vibert, 2007) contributing to the EU's perceived democratic deficit (Curtin and Dehousse, 2012). Accountability has been proposed as a solution to these democratic legitimacy problems, offering the potential to reconcile credibility (based on the independence and expertise of EU agencies) with democratic control (Majone, 1999). If the EU accountability landscape, with its variety of account‐holders, can provide sufficient checks‐and‐balances on the powers of EU agencies (Curtin, 2007), EU agencies could – to paraphrase Moe (1987, p. 291) – be under control even though no one controls them. At the end of the day, this will depend on how the EU account‐holders de facto execute account‐holding activities vis‐à‐vis EU agencies. By empirically examining the account‐holding intensity that EU agencies are subject to, this article empirically informs these discussions, providing us with a better understanding of whether or not EU agencies are the ‘hierarchy beaters’ (Everson, 1995) that they have been made out to be.

This article makes both theoretical and empirical contributions to scholarship on the accountability of EU agencies. Theoretically, it presents and applies a novel conceptualization of account‐holding intensity as both the *frequency* and the *diligence* with which account‐holding activities are executed. Empirically, the article is ambitious in scope, focusing on the account‐holding intensity with which six major institutional EU account‐holders execute account‐holding activities vis‐à‐vis 39 EU agencies. This amounts to a comprehensive empirical overview of the EU accountability landscape.

## I Conceptualizing Account‐Holding Intensity: Frequency and Diligence

Academic consensus has in recent years consolidated around a definition of accountability as ‘a relationship between an actor and a forum, in which the actor has an obligation to explain and to justify his or her conduct, the forum can pose questions and pass judgement, and the actor may face consequences’ (Bovens, 2007, p. 450). Accountability is defined here as a relational mechanism, and as such it is understood that the behaviour of (at least) two parties influence the effectiveness of accountability practices: the account‐holder(s) and the account‐giver(s) (henceforth also referred to as *forums* and *actors*, respectively).

However, despite the relationality of accountability, the behaviour of account‐holders has been undertheorized, with accountability literature often assuming that forums are motivated and willing to prevent ‘actor drift’ (Schillemans and Busuioc, 2015). One undertheorized aspect of account‐holding behaviour is the *intensity* with which forums discharge their account‐holding responsibilities. This despite empirical studies showing considerable variation in this regard, with many studies describing forums that neglect their account‐holding tasks (see Schillemans and Busuioc, 2015).

In this article, we define the concept of *account‐holding intensity* as a combination of the *frequency* and the *diligence* of account‐holding activities. With *frequency*, we refer to the *quantity* of account‐holding activities. This includes how often forums ask for reports or other types of performance information, how often they ask (follow‐up) questions and provide feedback about actor operations, how often they participate in meetings or hearings, how often they voice praise or criticism, or how often they use available control mechanisms (for example budget allocation, renewals/dismissal of executive's mandate, or changes to institutional tasks and mandates). With *diligence*, we refer to the *quality* with which these account‐holding activities are executed. That is, how thoroughly forums read information and reports that they receive, how actively they participate in meeting and hearings, the extent to which their questions and feedback are relevant to actors' operations and pertinent to the accountability processes in question, and the extent to which the praise/criticism and sanctions/rewards that they assign are justified by actors' conduct and performance.

Concrete examples of previous studies that are (implicitly) focused on what we refer to as frequency of account‐holding, concern for example how often parliamentary written questions are asked (Font and Perez Duran, 2016); how often different agencies are subject to ministerial contacts (Hogwood *et al*., 2000); how often performance measurement information is used (Pollitt, 2006); and how often various control mechanisms are used (Thatcher, 2005). Previous studies that are (implicitly) concerned with what we refer to as diligence have, in turn, reported about management board members who do not carefully process information that they receive, failing to notice missing pages in documents sent to them, and filling in travel reimbursement forms during account‐holding meetings (Busuioc, 2013); parliamentarians whose questions ‘do not amount to proper scrutiny’ (Kluger Dionigi, 2020, p. 91) as the questions do not address the issue or actor under scrutiny; or supervisors who do not want to take action against blatant errors or falsifications in the reports of service‐providing organisations (Dicke, 2002, p. 461).

We consider both frequency and diligence to be integral parts of the intensity of account‐holding. In order to hold to account with high intensity, a forum must engage in account‐holding practices (namely, frequency). However, just going through the motions of account‐holding is not enough as ‘frequent interactions do not necessarily translate into “more accountability”’ (Maricut‐Akbik, 2020, p. 1211). Intense account‐holding implies not only that account‐holding activities are executed with high frequency, but also with high diligence. Vice versa, the overall accountability pressure that an actor experiences from a diligent forum is still limited if the forum in question very rarely executes any account‐holding activities.

## II The Multiple Forums of EU Agencies

Accountability relationships in the EU are diverse and pluralistic, with multiple principals and non‐principal forums having responsibilities to hold EU agencies accountable. As such, the EU accountability landscape provides a prime opportunity to map the account‐holding intensities of different forums. In this study, we examine the account‐holding intensity of six major institutional EU account‐holders: the European Parliament, the European Commission, the Council of the European Union, Management Boards, the European Court of Auditors, and the European Ombudsman. We draw on extant literature on EU agencies and their accountability relationships in order to gain insights into what patterns we might expect about the frequency and the diligence with which these different accountability forums execute account‐holding tasks. This allows us to engage with relevant previous literature, aiding our discussion about what this study tells us about the EU accountability landscape. Consequently, we will return to these expected patters at the end of this article, comparing them to the patterns that we observe in our study.

### The European Parliament

With regards to the European Parliament, Busuioc (2013) has noted that while the EP has successfully fought for extended rights to receive information from EU agencies, there seems to be considerable variation in actual parliamentary interest in agencies' work. For example, some agencies had no contact with their parent EP committee, and some agencies experienced poor attendance from the parliamentarians (MEPs) in hearings. Similarly, Font and Perez Duran (2016) found that three agencies received almost half of all parliamentary written questions to EU agencies during the seventh legislature. Additionally, when the EP does engage in account‐holding practices, the quality of its performance as an account‐holder has been questioned. For example, Kluger Dionigi (2020, p. 91) has concluded that ‘[t]he majority of the questions asked [by MEPs] during the economic dialogue do not amount to proper scrutiny’. Furthermore, in relation to the EP's account‐holding of the European Central Bank under the Single Supervisory Mechanism, Maricut‐Akbik (2020, p. 1207) found that ‘more than a fifth of all hearings is wasted on questions that are outside the scope of accountability’. Thus, *we expect the European Parliament to hold EU agencies to account with varying frequency and with low diligence*.

### The Council of the European Union

Similarly to the European Parliament, the Council of the European Union has also been described to be selective in its account‐holding, with its account‐holding attention changing with the rotating EU presidencies and their varying political agendas (Busuioc, 2013). The attention that the Council pays to agencies has furthermore been found to be larger for agencies dealing with particularly politicized issues (Egeberg and Trondal, 2011). As such, expectations with regards to the account‐holding of the Council are similar to that of the EP. That is, *we expect the Council of the European Union to hold EU agencies to account with varying frequency and with low diligence*.

### The European Commission

Relevant Directorate‐Generals (DGs) of the European Commission have been found to have actively and successfully positioned themselves as the ‘parents’ of the EU agencies (Egeberg *et al*., 2015). By way of informal meetings, phone calls, and emails, the EC has been described as the EU forum that is in closest contact with EU agencies (Egeberg and Trondal, 2017), and EU agencies have in turn been reported to take the concerns of the EC into consideration to a higher extent than that of any other EU institution (Egeberg and Trondal, 2011). As such, it has been suggested that the EC has more control over EU agencies than what is derived from formal‐legal statutes (Egeberg *et al*., 2012). Consequently, *we expect the European Commission to hold EU agencies to account with both high frequency and diligence*.

### Management Boards

The Management Boards of EU agencies have been described as ‘the main and most direct confines on the grant of authority of the agencies and their directors’ (Busuioc, 2012, p. 719). To this end, most MBs have regularly scheduled meetings (often quarterly) with the purpose of monitoring and overseeing the performance of their EU agency. However, there are empirical findings demonstrating that while ‘some board delegations (…) are well prepared and on top of their game, an overwhelming number are not the vigilantes they are formally mandated to be’ (Busuioc, 2012, p. 732). Some MB members have been found to not seek detailed information, instead being content with receiving general and descriptive reports of agency performance, which many delegations furthermore reportedly do not read carefully. As a consequence, some MB members have been found to attend meetings poorly prepared, often not actively participating, seemingly lacking an interest in overall agency performance (Busuioc, 2012, 2013). Thus, *we expect the Management Boards to hold EU agencies to account with moderate frequency, and with low diligence*.

### The European Court of Auditors

The European Court of Auditors has been described as performing its account‐holding tasks (namely, audits) vis‐à‐vis EU agencies diligently (Busuioc, 2013). Furthermore, the ECA has also been found to have become increasingly active during its lifespan, publishing an increasing number of audit reports (Wille, 2016). The ECA's increased activity has been noted to be largely driven by an increase in so‐called ‘special reports’: reports focused on specific issues. It has been argued that the ECA is showcasing its diligence by going beyond its formal account‐holding obligations in this way (Tidå, 2021). Consequently, *we expect the ECA to hold EU agencies to account with both high frequency and diligence*.

### The European Ombudsman

Similarly to the European Court of Auditors, the European Ombudsman has also been described to perform its account‐holding activities vis‐à‐vis EU agencies diligently (Busuioc, 2013), exercising its accountability powers with credibility, creativity, visibility and impact (Wille and Bovens, 2020). Like the ECA, the EO has been noted to have increased its account‐holding activity during its lifespan, launching an increasing number of investigations (Wille, 2016). The EO has furthermore been described to deploy an increasingly ‘intensive’ style of review (Harlow and Rawlings, 2007, p. 557), increasing its focus on so‐called ‘strategic inquiries’, launching own‐initiative investigations into specific administrative issues. As such, it has been argued that the EO has become an impactful stakeholder for increasing accountability and transparency in the EU (Kostadinova, 2015). Consequently, *we expect the EO to hold EU agencies to account with both high frequency and diligence*.

## III Data and Method

In order to answer the research question, we opted for a *complementary* mixed‐methods approach, in which we seek to elaborate, enhance, illustrate and clarify the results of one method, with the results of another method (Greene *et al*., 1989). Concretely, we rely primarily on quantitative survey data and qualitative interview data collected from top‐level managers of EU agencies from October 2019 to March 2020. The quantitative survey data allows us to estimate the intensity with which different forums perform their account‐holding tasks, while the qualitative interview data provides detailed insights into what might explain why certain forums are more active and diligent than others.

Interviewing and surveying account‐givers (in this case EU agencies) comes with advantages compared to collecting data from account‐holders, which might be affected by self‐assessment bias. It also has advantages compared to unobtrusive account‐holding data, which is by nature restricted to formal accountability practices, thus not capturing potentially influential informal account‐holding. To further improve the validity of the findings, we do however triangulate the survey and interview data gathered from agency directors and senior managers with additional data from in‐depth interviews with account‐holders, as well as a variety of unobtrusive account‐holding data including: written questions from the European Parliament; meeting minutes from the Council of the European Union; special reports from the European Court of Auditors; and cases opened by the European Ombudsman.

### Survey Data

The online survey was disseminated via email to the 220 executive directors, deputy directors, administrative directors, and operational directors of all 45 members of the EU Agencies Network. 1 We deliberately targeted top‐level managers, as they are usually the most involved in external accountability processes and have the most direct contact with institutional account‐holders. As Yang (2009) writes: ‘organizational reporting is usually a decision made by a collective of top managers or the leadership team’ (p. 84). As such, top‐level managers have unique knowledge about the operations of accountability processes and serve as key informants for our study. In total, 75 respondents (34.1 per cent) from 39 EU agencies (86.7 per cent) completed the survey (see Table 1).

**Table 1:** Organizational Position of Survey Respondents

*N*	*Organizational position*				
	*Executive*	*Deputy*	*Administrative*	*Operational*	*Other*
75	18	4	19	30	4

In line with our conceptualization of account‐holding intensity, the survey questions (Table 2) were designed to capture both *frequency* and *diligence*. The questions also capture three accountability phases: *information*, *discussion* and possible *consequences*. These three phases follow directly from the definition of accountability that we adhere to in this study (Bovens, 2007). Answers to all questions were given on five‐point scales.

**Table 2:** Survey Questions

*Accountability phase*	*Question*	*Answer option*				
		*1*	*2*	*3*	*4*	*5*
Information	Q1 (frequency). *On average, how often is your agency asked to report or present its work to representatives of the following actors?*	Never	Once per year or less	2–6 times per year	7–11 times per year	12 or more times per year
	Q2 (diligence). *Please indicate to what extent you agree with the following statement for each actor: Representatives of this actor thoroughly read what your agency reports about its work*.	Strongly disagree	Disagree	Neither agree nor disagree	Agree	Strongly agree
Discussion	Q3 (frequency). *How often do representatives of the following actors provide feedback when they receive information from your agency?*	Never	On rare occasions	About half the time	Often	Always
	Q4 (diligence). *Please indicate to what extent you disagree or agree with the following statement for each actor: Feedback from representatives of this actor contribute to relevant discussions about the work of your agency*.	Strongly disagree	Disagree	Neither agree nor disagree	Agree	Strongly agree
Consequences	Q5 (frequency/diligence). *How likely are representatives of the following actors to use available rewards/sanctions against your agency?*	Very unlikely	Unlikely	Neither likely nor unlikely	Likely	Very likely

For the information phase, we asked how often the account‐holders make reporting requests (frequency), and the extent to which the account‐holders thoroughly read the reports (diligence). We asked respondents this question to measure diligence in the information phase, as the thoroughness with which forums' process information provided by the actor is of crucial importance to their abilities to meaningfully participate in follow‐up account‐holding activities (for example Busuioc, 2013, pp. 87–9).

For the discussion phase, we asked how often account‐holders provide feedback on information that the agency has provided (frequency), and the extent to which this feedback is relevant (diligence). We asked respondents this question to capture diligence in the discussion phase, as irrelevant questions have been identified in previous studies as a detriment to the quality of account‐holding (for example Kluger Dionigi, 2020).

For the phase of possible consequences, we asked only one question – designed to capture aspects of both frequency and diligence – about the likelihood of account‐holders using available rewards/sanctions against the agency. We chose to do this following pilot interviews where respondents indicated that they were not in a position to judge the extent to which sanctions/rewards are well‐justified (diligence). We therefore pose only one question, asking about *likelihood* instead of pure frequency, thus aiming to indirectly capture also aspects of diligence by triggering respondents to think about how likely account‐holders are to respond to their poor/good performances with suitable sanctions/rewards.

For our analysis, we consider answers on the low‐end of the scales (1–2) as indicative of low account‐holding frequency/diligence, answers on the mid‐point of the scales (3) as indicative of moderate account‐holding frequency/diligence, and answers on the high‐end of the scales (4–5) as indicative of high account‐holding frequency/diligence. Answers that are spread out across answer categories, with a large proportion located on both the low‐ and the high‐end of the scales, are considered indicative of account‐holding frequency/diligence being of high variance between agencies.

It is important to note that low‐intensity account‐holders are not per definition neglecting their account‐holding responsibilities, nor are high‐intensity account‐holders per definition putting an undue burden on EU agencies. Different forums have different account‐holding tasks and obligations, which require different account‐holding intensities to fulfil. As such, directly comparing the account‐holding intensities of different forums, without accounting for their different roles and responsibilities, would be misleading. This is why it is valuable to complement the quantitative survey data with qualitative interview data. By combining these types of data, we can properly contextualize the measured account‐holding intensities, helping us to understand why a specific forum showcases low/high account‐holding frequency/diligence, and in extension whether or not it could be a source of an accountability deficit or overload.

### Interview Data

We conducted 15 in‐depth semi‐structured interviews with agency representatives (Appendix II, Table 1). Interviewees were asked to fill in the survey before the interview. We asked interviewees to expand on a selection of survey responses that we considered particularly interesting, being either typical or atypical to observed patterns in the survey data that had already been collected.

Interviewees were approached following a purposive sampling strategy. We aimed to maximize the breadth of agencies represented in our data. Concretely, interview requests were sent to executive and deputy directors of 23 agencies. Ten executive directors, and one deputy director, accepted our invitation. In four cases, we were referred to other people within the organization with knowledge relevant to our study whom we then proceeded to interview.

In order to compare the agency perspective with the perspectives of some of the account‐holders themselves, we also used data from interviews with nine auditors, principal managers, and national members of the European Court of Auditors, one member of the European Parliament, and one senior official of the directorate‐general Economic Affairs and Competitiveness at the Council of the European Union (Appendix II, Table 2).

Interviews were conducted face‐to‐face, via video call, or over the phone, and lasted approximately one hour. All interviews were recorded and transcribed with the informed consent of the interviewees. Interviews have been assigned a number (Agency #1 – Agency #15; ECA #1 – ECA #9; EP #1; Council #1) in order to preserve the anonymity of interviewees. The interview transcripts were coded in Atlas.ti.

### Unobtrusive Data

Unobtrusive account‐holding indicators were collected from a variety of public sources. The indicators were collected from (i) 30,965 European Parliament written questions addressed to the European Commission 2 during the eighth parliamentary term (2014–19); (ii) 322 Council of the European Union meeting minutes (2015–19); (iii) 148 European Court of Auditors special reports (2015–19); and (iv) 929 cases opened by the European Ombudsman (2015–19) (Appendix III, Tables 4–7). From these documents we counted: (i) how many times different agencies were the subject of written EP questions; (ii) how many Council meeting minutes addressed the different agencies; (iii) in how many ECA special reports EU agencies were (one of) the main auditee(s); and (iv) how many times different agencies were subject to cases opened by the EO (Appendix III, Table 3).

## IV Zooming in: The Account‐Holding Intensities of the EU Forums

Before we take a consolidated look at the overall view of the account‐holding intensity in the EU accountability landscape, we will zoom in on the specific forums. Doing so, we use the interview data to contextualize the survey results, highlighting the forum‐specific behaviours and institutional characteristics that drive their account‐holding.

### The European Parliament: Politicians with Budget Discharge Authority

**Figure 1:**
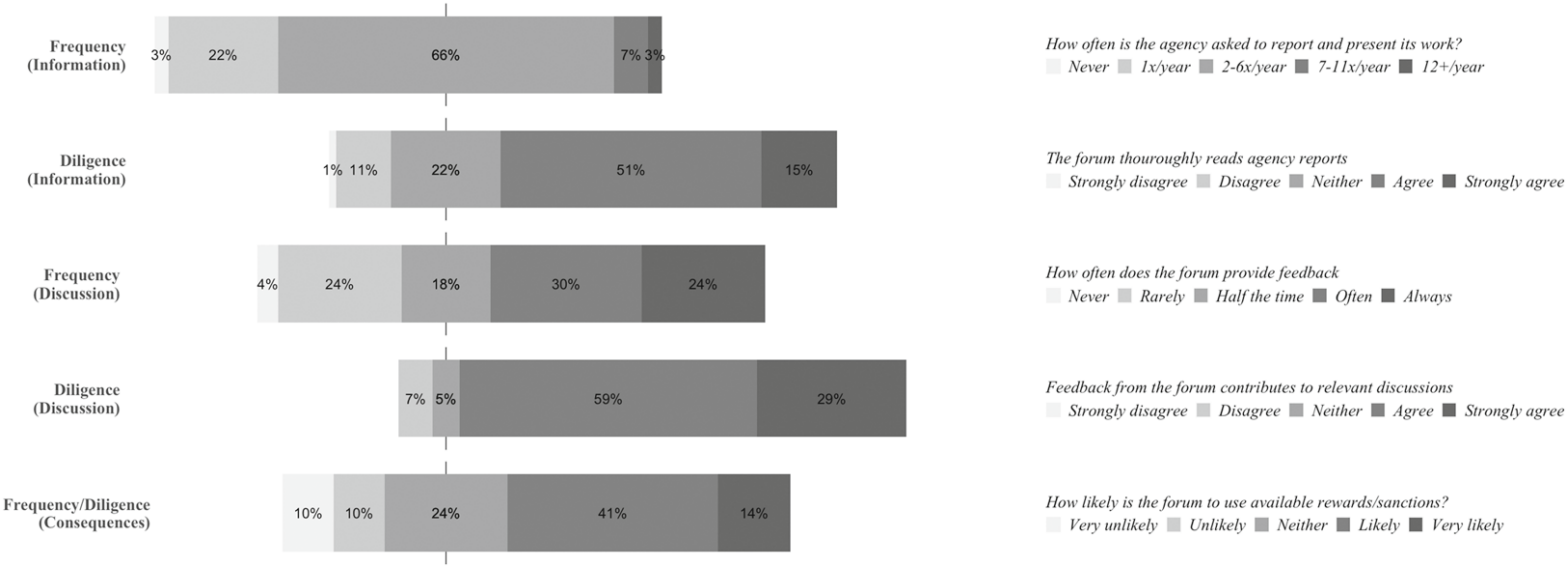
The Account‐Holding Intensity of the European Parliament *Source*: Authors' survey.

Examining the answers to the survey questions relating to the European Parliament, we observe that the frequency with which the EP asks agencies for information is moderate. Only seven respondents (10 per cent) place the frequency of the EP's requests for information on the high‐end of the scale (Figure 1). This is quite consistent with how EP written questions during the eighth parliamentary term (2014–19) were distributed among agencies. Almost all agencies received some EP questions, with only two agencies not receiving any questions during this five‐year period. However, high‐frequency account‐holding is reserved for only a few agencies, as more than half (51.5 per cent) of all the EP's written questions to agencies were directed at only three agencies: EFSA, Europol, and Frontex (Appendix III, Table 3). In addition, we observe a large variance in respondent answers with regards to the frequency of feedback from the EP (Figure 1). This indicates that the EP's account‐holding frequency varies considerably between agencies.

While the survey data indicates that the EP's account‐holding frequency is moderate in the information phase and of high variance in the discussion phase, its account‐holding diligence is consistently reported as high (Figure 1). Noticeably, some interviewees provide statements that seem to contrast this image of the EP as a diligent account‐holder. For example, interviewees note that ‘the European Parliament sometimes lacks expertise and good understanding’ (Agency #13), being unaware about important aspects of agencies' operations (Agency #8, #15), asking questions about issues outside agency mandates (Agency #15), or asking questions which have already been answered in available reports (Agency #13, #15).

However, many interviewees are lenient in their assessments of the MEPs account‐holding on these aspects, referring to the complex technical nature of the agencies' work. To illustrate: ‘you would really need an expert to have a proper professional discussion [about the work of the agency], and that's not, I think, the task of the Parliament’ (Agency #1). The interview data furthermore indicates that the relevance of EP questions and feedback, instead of being based on technical expertise, has its source in democratic representation (Agency #2, #5, #6, #14). MEPs are considered to be the EU account‐holders ‘nearest to the citizens’ (Agency #6). As such, account‐holding from the EP ‘forces us to always think: Okay, what we do, to what extent does it impact the daily life of the citizens?’ (Agency #14).

Consistent with the results of the survey data, the interview data also suggests that the EP is a forum whose account‐holding frequency varies between agencies. Interviewees provide similar explanations for why this is the case. Namely, that MEPs are sensitive to political circumstances. One interviewee summed this up concisely, stating that ‘with the Parliament it's very simple. (…) they [MEPs] are operating and thinking all from this political perspective’ (Agency #7). As such, the account‐holding frequency of the EP vis‐à‐vis a particular agency is highly dependent on agency salience. To illustrate, one interviewee who reported that its agency receives a lot of account‐holding attention from the EP, explained this accordingly:

the political attention for [Agency] is higher than possibly for every other of the EU agencies. Because [area of competence], it's an issue that is so emotional that the attention from the public is so high. (Agency #4)

Another recurrent theme in the interview data concerns the EP's role as a budget discharge authority. Some interviewees explicitly describe the budget discharge process as the ‘main’ (Agency #13) and the ‘ultimate’ (Agency #12) accountability process in the EU, being ‘the most impactful decision that can be made’ (Agency #4). The frequency of the EP's activities in the discharge process is described as consistent across agencies. Almost all agencies annually have to answer a questionnaire and participate in a budget discharge hearing in the EP's committee on budgetary control (CONT). However, the extent with which the MEPs follow up on the information they receive through these practices is described to vary. Here, variations are driven not by salience but by the findings of the European Court of Auditors, whose reports are, ‘the source number one’ (EP #1) for the EP in the budget discharge process. This is also recognized by agencies. To illustrate: ‘the parliament bases its decision on what is in the Court of Auditors reports’ (Agency #2). A positive report from the ECA means less intense scrutiny: ‘if the Court [of Auditors] tells our Parliament and Council: “[Agency] has no major findings”, the cake is nearly eaten’ (Agency #1). On the other hand, a negative report from the ECA will lead to more intense scrutiny: ‘If the Court of Auditors says: “There is a major issue. There is a critical recommendation.” Then it's a very strong signal, and then you have to explain yourself [to the EP] in the discharge’ (Agency #2).

### The Council of the European Union: Representatives of National Interest

**Figure 2:**
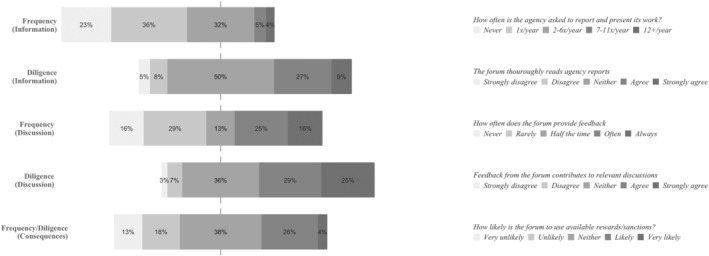
The Account‐Holding Intensity of the Council of the European Union *Source*: Authors' survey.

The survey results related to the account‐holding frequency of the Council of the European Union display high variance (Figure 2). This indicates that the Council is a frequent account‐holder for some agencies, while for other agencies it is not. Corroborating this picture, we see that four agencies (EFSA, Eurojust, Europol and Frontex) dominate the Council's attention: 152 out of the 324 (46.9 per cent) times that EU agencies are mentioned in the minutes of a Council meeting (multiple mentions of the same agency in one meeting counted only once) refer to one of these four agencies (Appendix III, Table 3).

Similarly to the European Parliament, interviewees view salience as an explanation for why the frequency of the Council's account‐holding varies. As such, one interviewee who reported that its agency does not receive much account‐holding attention from either the EP or the Council, explained this accordingly:

We're not even in that political, I would say, discussion. So our core [task] is very, well you could say it's not interesting for people who are not in the business. (…) So, we are very uncontroversial. We rarely are spoken of in the media. Because we do not create scandals or all these kinds of things. So, I think for that reason, the Council and the Parliament, they are less asking [for] information on what we are doing. (Agency #10)

Interviewees furthermore note that the account‐holding intensity of the Council is subject to change over time. Such changes can occur due to national elections leading to changes in compositions of member state governments (Agency #12), or a change in the rotating Council presidencies (Agency #12), whose focus is ‘driven in the end by their national interests.’ (Agency #7). In addition, the Council's account‐holding intensity is also affected by external developments, as various crises and emergencies force Council presidencies to focus on agencies dealing with these issues (Agency #7).

Yet another similarity between the Council and the EP is that the Council also has a role – albeit a smaller one – in the budget discharge process, providing the EP with a (non‐binding) recommendation on the EP's discharge decision. Similarly to the EP, the Council ‘bases everything on the Court of Auditors’ material’ (Council #1). In other ways, the account‐holding behaviours of the EP and the Council as budget discharge authorities differ, as the Council is described to have very limited interactions with the majority of agencies. Some interviewees (Agency #3, #12, #14) even note that their agencies have never received an invitation to a budget discharge meeting in the Council. To illustrate: ‘we have never been invited – or consulted or requested about anything about our activities – from the Council in the context of the discharge’ (Agency #14).

### The European Commission: An (Inter)Dependent Parent

**Figure 3:**
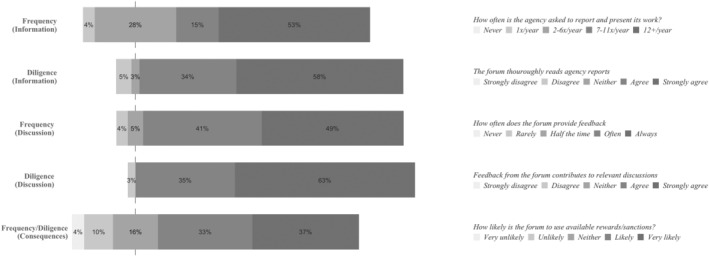
The Account‐Holding Intensity of the European Commission *Source*: Authors' survey.

Survey results indicate that the European Commission is a very high intensity account‐holder. For all survey questions, a majority of respondents place the EC on the high‐end of the answer scale (Figure 3).

The interview data corroborates the picture of the EC as a high‐intensity account‐holder, with many interviewees particularly highlighting that their agency has very frequent contacts with the EC (Agency #2, #7, #8, #9, #10, #11, #15). Worth noting is that the interviewees describe a lot of these contacts as informal:

We are constantly in contact with our counterparts in DG [X] in the Commission. By phone, in writing, by replying to questions, by bringing instances, by discussing with [them] the way we do things, and by giving them account of what we do. So [these] are all informal reporting situations (Agency #4).

Explanations from interviewees with regards to the high frequency of EC account‐holding often refer to the agencies and the EC being operationally close, with the specific tasks of the agencies often depending on what its ‘parent DG’ (Agency #1, #3, #4, #12) expects and asks them to do. One interviewee stated that the agency considers itself ‘the operational arm of the European Commission (Agency #11)’. To illustrate further:

Our parent DG of the Commission basically sends us all the time questions. And we are supposed to develop our work to respond to those questions. So, we do not invent the work that we do, but we actually perform it in response to questions that we receive from our policy master (Agency #4).

Furthermore, the EC is in turn described as also being dependent on EU agencies to successfully fulfil its own (regulatory) tasks. To illustrate: ‘many of these tasks that we're legislated to do end up in providing information which the Commission uses to write its regulations or take its decisions’ (Agency #8). In other words, there seem to be mutual operational interdependencies between the EC and the agencies driving their close contacts.

### Management Boards: Semi‐internal Account‐Holders

**Figure 4:**
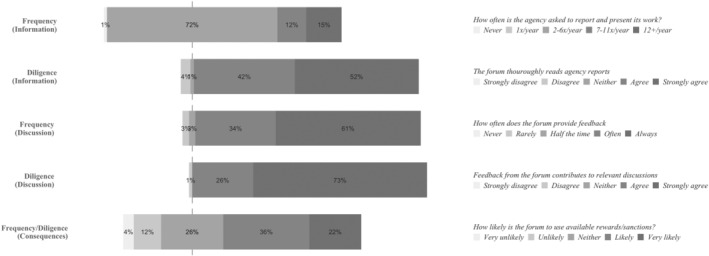
The Account‐Holding Intensity of Agency Management Boards *Source*: Authors' survey.

The survey results for the agency Management Boards indicate that they are high intensity account‐holders. Apart from the frequency of account‐holding in the information phase, which is more moderate, respondents place MBs on the high‐end of the answer scales (Figure 4).

The interview data corroborates that MBs are high‐intensity account‐holders. To explain why, many interviewees refer to the organizational proximity between the agency and its MB. Being semi‐internal account‐holders of the agencies, MBs are directly responsible for agency conduct and performance: ‘Legally, every decision on any aspect of our operation is decided by the Management Board.’ (Agency #2). This increases MB members' incentives to intensively monitor agency performance. To illustrate:

In our basic regulation, it states that they [the management board members] should monitor the activities of the [Agency]. So, when we do the reporting and when we do tell them what we are going to do, if they are not happy about that, they react immediately. And we discuss on how to go forward. (Agency #10).

Consequently, many interviewees describe MB meetings as intense accountability occasions, in which the work and performance of the agencies are diligently discussed. These meetings are regularly scheduled, with most MBs meeting quarterly: ‘We have a number of KPIs – it is quite a long list of KPIs – on which we report every three months to the management board the way we actually perform’ (Agency #4).

### The European Court of Auditors: Professionals with a Nitty‐Gritty Process

**Figure 5:**
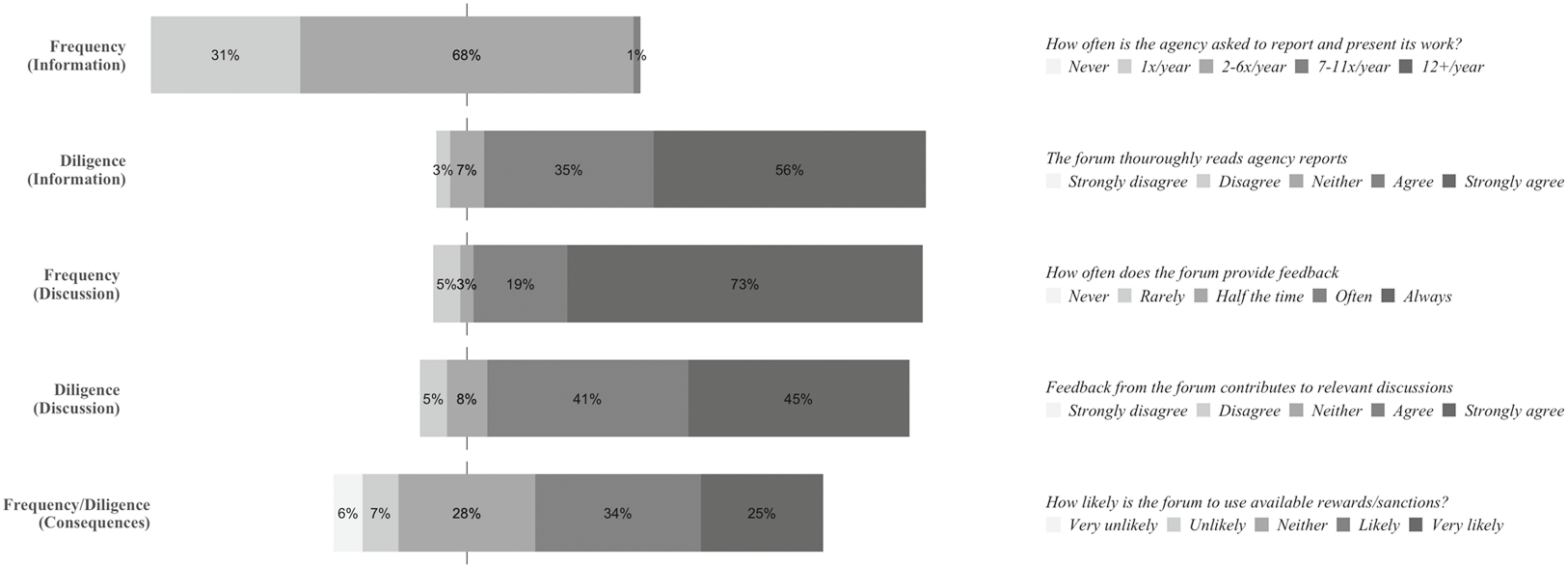
The Account‐holding Intensity of the European Court of Auditors *Source*: Authors' survey.

The survey results for the European Court of Auditors show that the frequency with which the ECA is asking agencies for information is moderate to low. However, the survey results also indicate that the ECA makes up for this by being a very high‐intensity account‐holder in all other account‐holding aspects (Figure 5).

An explanation for the moderate frequency with which the ECA is found to ask agencies for information could be that agencies' contacts with the ECA almost exclusively relate to concentrated oversight periods pertaining to the annual audits. While the ECA has published an increasing number of special reports in recent years, agencies are rarely subject to these reports. Among the 148 special reports published by the ECA 2015–19, only seven reports (4.7 per cent) concerned at least one EU agency as a main auditee (Appendix III, Table 6).

The (annual) audit process is however described as very intense. The ECA almost always follow‐up on their information requests, engaging the agencies in discussions and providing relevant feedback (Figure 5). In short, these audits are ‘quite a process of clearing facts, of understanding different viewpoints, of discussing the issues and trying to agree on common standpoints.’ (ECA #3). Oftentimes discussions take place not only once, but several times through a variety of both formal and informal interactions. In these interactions, the ECA is described as very diligent, being *‘*the one [account‐holder] that goes into the nitty‐gritty and the details’ (Agency #3). To illustrate:

they come here for a week to inspect our accounts or do surveys. But they are physically here for a week. And during the week, they have a lot of interactions with colleagues. Asking questions, but also then getting answers. We have a wrap‐up meeting after the audit, where they give us the preliminary findings that we then already can discuss. Then there are informal interactions, video conferences. And then there's a formal process afterwards. So there's really lots of dialogue (Agency #8).

### The European Ombudsman: Out of Sight, Out of Mind?

**Figure 6:**
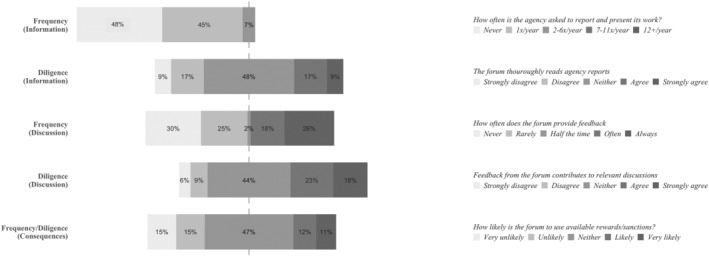
The Account‐Holding Intensity of the European Ombudsman *Source*: Authors' survey*.*

What stands out when examining the survey results of the European Ombudsman is its low account‐holding frequency in the information phase. 34 respondents (48 per cent) answered that their agency is *never* asked to report or present its work to the EO (Figure 6). A look at the number of cases opened by the EO concerning EU agencies 2015–19 corroborates this notion, as 18 agencies (40.0 per cent) were not subject to any EO investigation. In addition, for another ten agencies (22.2 per cent), only one opened case during this five‐year period concerned them (Appendix III, Table 3).

The interview data is consistent with the survey findings, as interviewees consistently note that they very rarely have contact with the EO. Interviewees recurrently explain this by referring to the complaint‐driven nature of the EO's account‐holding tasks. Agencies' contacts with the EO are largely dependent on whether or not the EO receives complaints about the agency. Multiple interviewees state that their agency is seldomly the target of such complaints (Agency #2, #3, #10, #15). Consequently, their contacts with the EO are limited: ‘We basically have no contact with the European Ombudsman, which is also explainable because the Ombudsman also only acts when the Ombudsman is asked by somebody’ (Agency #2).

It should be noted that in addition to its complaint‐based investigations, the EO can launch strategic inquiries on its own initiative. However, also in this regard interviewees report very limited interactions: ‘Sometimes, I think we have gotten a questionnaire from them or something. But it's really, really rare.’ (Agency #10).

## V Zooming out: Account‐Holding Intensity in the EU Accountability Landscape

Our results are consistent with many of the patterns that we expected to find. For example, the results indicate that the European Commission has successfully claimed the role as ‘parent’ – or in the words of our interviewee: ‘policy master’ – of EU agencies through intensive informal contacts (see also Egeberg and Trondal, 2011, 2017). Hence, this study provides further support to the argument that EU agencification allows the EC to expand its influence during the post‐delegation phase (Kelemen and Tarrant, 2011). Furthermore, the account‐holding frequencies of the European Parliament and the Council of the European Union is found to vary between agencies, as the focus of these political account‐holders is influenced by salience, and political/national interests (see also Busuioc, 2013; Egeberg and Trondal, 2011; Font and Perez Duran, 2016). Additionally, results indicating that the European Court of Auditors is a diligent account‐holder (see also Busuioc, 2013) also conforms with expected patterns.

Yet, we find some deviations from the patterns we expected. For example, in contrast to findings from prior studies (for example Busuioc, 2012, 2013), Management Boards are reported to be active and diligent account‐holders. This finding could be interpreted in the light of studies of the MBs of national agencies, which show that even though MBs tend to remain flawed as account‐holders, their performance improves with time as they ‘grow’ into their roles (Schillemans and Bovens, 2019, p.197). This should increase optimism that MBs can effectively serve as linchpins between EU governance and democratic institutions at the national level (see Buess, 2015). Additionally, the European Ombudsman is, in contrast to findings from prior studies (for example Wille and Bovens, 2020), found to be a largely passive account‐holder. However, our interview findings indicate that this is a function of its institutional nature. While the EO can launch own‐initiative investigations, its main role is that of investigating complaints. The EO's diagnosed lack of account‐holding frequency may thus simply be a consequence of most agencies very rarely being the subject of complaints to the EO.

Furthermore, an important nuance pertaining to the EP findings deserves to be explicitly addressed. The survey results indicate that the EP is a diligent account‐holder. Simultaneously, there is evidence in the interview data indicating a lack of diligence (that is, account‐holding reflecting a lack of expertise, knowledge and understanding of agency operations) that is similar to how the EP has been found to execute its account‐holding tasks in prior research (for example Kluger Dionigi, 2020; Maricut‐Akbik, 2020). This points towards the EP being diligent about other aspects than operational and technical details, as interviewees instead highlight how the EP's account‐holding pushes them to think about how their work impacts EU citizens.

The image that emerges when we zoom out from these forum‐specific results to take a consolidated look at the overall EU accountability landscape is that of a complex ecology, where different forums execute account‐holding activities with considerably different account‐holding intensities, as they are driven by different institutional logics (Table 3).

**Table 3:** Overview of Results

*Forum*	*Expected patterns*		*Observed patterns*		
	*Frequency*	*Diligence*	*Frequency*	*Diligence*	*Main driver of account‐holding*
EP	Varying	Low	Varying	High	Political/electoral interests and salience
Council	Varying	Low	Varying	Moderate	National interests and salience
EC	High	High	High	High	Operational (inter)dependence
MB	Moderate	Low	High	High	Organizational proximity
ECA	High	High	Moderate	High	Professional processes
EO	High	High	Low	Moderate	Complaints

## Conclusions

The article has sought to make both theoretical and empirical contributions to scholarship on the accountability of EU agencies. Theoretically, the article has contributed with a novel conceptualization of account‐holding intensity as both the *frequency* and the *diligence* with which account‐holding activities are executed. This constitutes a first step towards a framework for measuring both the *quantity* and the *quality* of account‐holding in a systematic manner. Our analysis of the accountability landscape of EU agencies has illustrated the analytical potential of the framework by allowing us to map the account‐holding intensity of a variety of different forums with detail and precision. Nonetheless, there is room to further refine this framework – both conceptually and operationally. Further research could, for example, focus on exploring how forums motivate their decisions to (not) impose sanctions or rewards in order to better understand how frequency and diligence play out in the consequences phase. Moreover, developing a validated survey scale of account‐holding intensity would make a great contribution towards measuring the concept of account‐holding intensity with increased reliability.

Empirically, the consolidated image of the EU accountability landscape sketched in this article constitute good news for those concerned with the (potential) accountability deficits of EU agencies, and how this could contribute to the EU's (perceived) democratic deficits (for example Curtin and Dehousse, 2012). Some forums have been found to be more active and diligent than prior studies would indicate, suggesting that forums, over time, can grow into their account‐holding roles. Furthermore, while the frequency of account‐holding for the majority of forums is either low (EO), moderate (ECA), or of high‐variance (EP and the Council), the diligence of account‐holding is for all forums moderate (the Council and EO) to high (EP, EC, MB, ECA). The high diligence observed should serve to temper concerns of accountability deficits which an analysis solely focused on frequency might have caused, as it indicates that the (vis‐à‐vis some agencies) less frequent forums have not abdicated their account‐holding responsibilities but are better characterized as being on ‘stand‐by’, acting in the face of complaints (EO) or increased salience (EP and the Council).

Furthermore, ‘selective’ account‐holding – as observed by the European Parliament and the Council of the European Union – has in prior studies been put forth as a possible source of legitimacy deficits in the EU due to the potential neglect of non‐salient agencies (for example Font and Perez Duran, 2016, p. 1363). Consistent with such concerns, the account‐holding frequency of the EP and the Council has in this study been identified to vary a lot between agencies, and interviewees have described salience as a main driver of their account‐holding. At the same time, our study also reveals that both the EP and the Council are highly responsive also to information from a full‐time account‐holder such as the European Court of Auditors. This is especially clear in the budget discharge process, where the EP (and to a lesser extent the Council) executes a very influential account‐holding function by relying heavily on information provided by the ECA. This indicates that the selective account‐holding of the EU's political account‐holders might be informed by balanced set of ‘fire alarms’ (McCubbins and Schwartz, 1984) that goes beyond public opinion and visibility. This identified ‘interdependency’ (Scott, 2000) is promising in that it indicates that professional institutional account‐holders such as the ECA could serve to temper the salience‐driven tendencies of political account‐holders. This result highlights the benefits of the broad perspective that this study undertakes – focusing on a variety of forums in the accountability landscape – allowing us to identify important interdependencies that can help compensate for the shortcomings of specific forums when looked at in isolation.

## List of Interviews

Agency #1–15. October 2019 – March 2020. Personal Interviews.

Council #1. December 2019. Personal Interview.

ECA #1–9. March – October 2019. Personal Interviews.

EP #1. October 2019. Personal Interview.

## Supporting information


**Data S1.** Supporting information.

## Footnotes

^1^
When we began data collection in October 2019, ELA, EPPO and EuroHPC were not yet members.

^2^
Written questions to EU agencies are addressed to the EC, which forwards the questions to the agencies and returns agencies' answers to the EP (Font and Perez Duran, 2016, p. 1351).
