# Supplementary material for: Account‐Holding Intensity in the EU Accountability Landscape: A Comprehensive Review of EU agencies' Institutional Accountability Relationships
Source: J Common Mark Stud. 2022 Jun 20;61(1):215–35. doi: 10.1111/jcms.13367 (PMC10084275; doi:10.1111/jcms.13367)
Supplement: Supplementary file 1 — Data S1. Supporting information. [file JCMS-61-215-s001.docx]

**Appendix**

**I: Survey Data**

*Within-Agency Variation*

Figure 1.1: Within-agency variation – EP


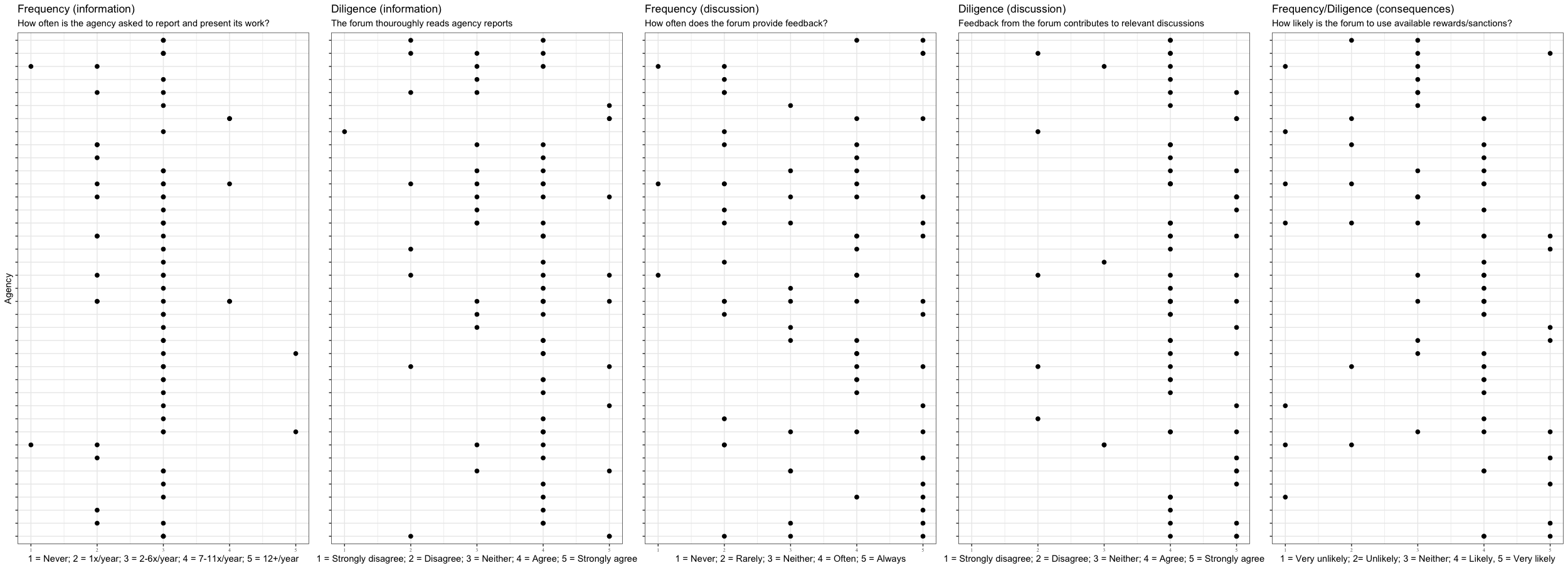


Source: Authors’ survey

Figure 1.2: Within-agency variation – Council

*
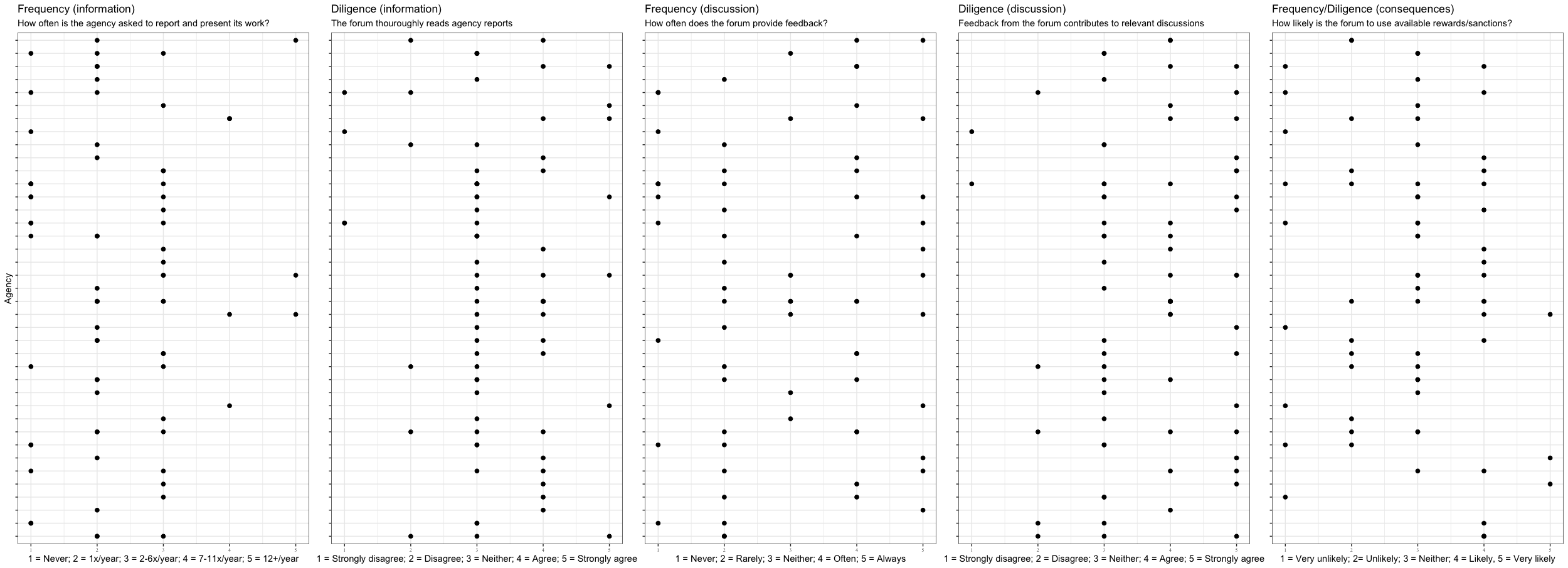
*

Source: Authors’ survey

Figure 1.3: Within-agency variation – EC

*
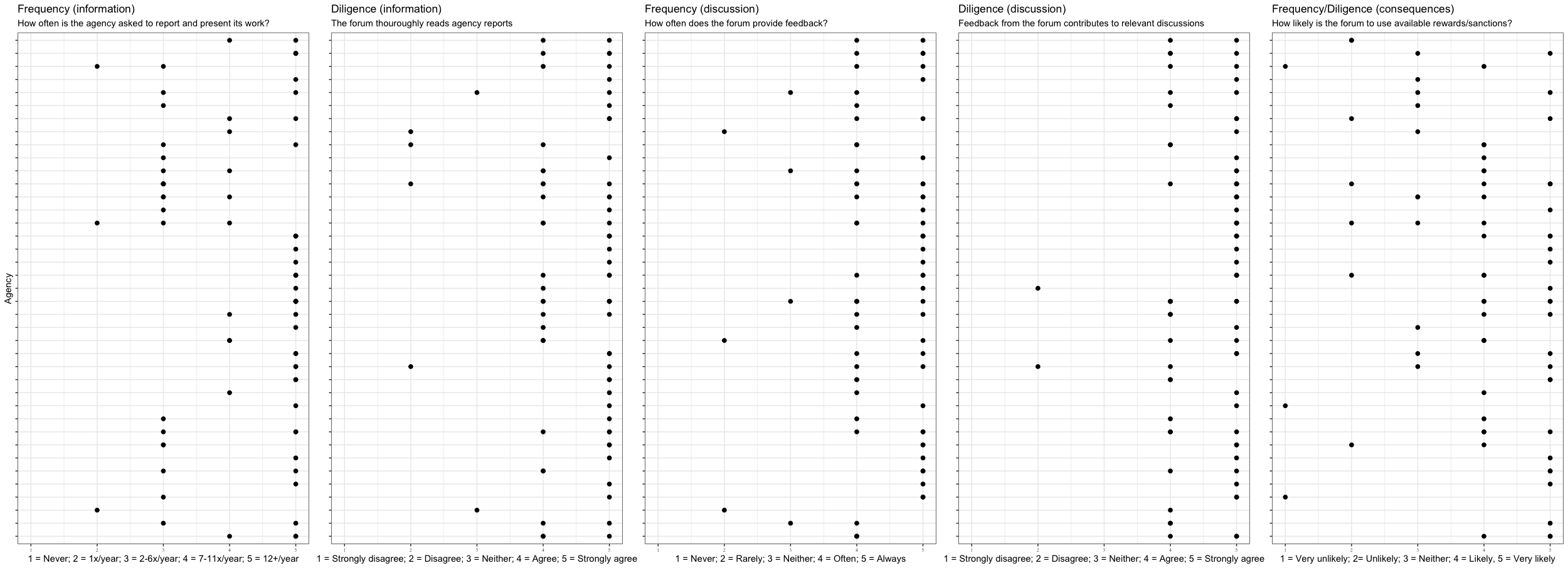
*

Source: Authors’ survey

Figure 1.4: Within-agency variation – MB


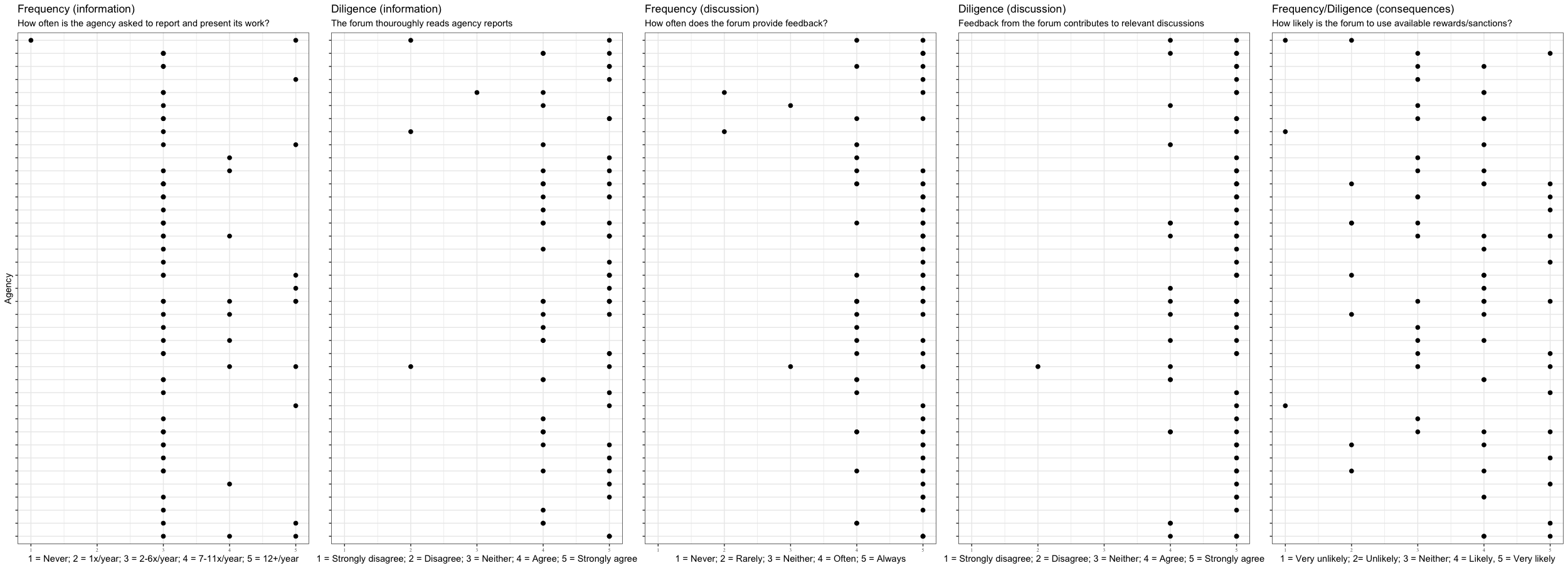


Source: Authors’ survey

Figure 1.5: Within-agency variation – ECA


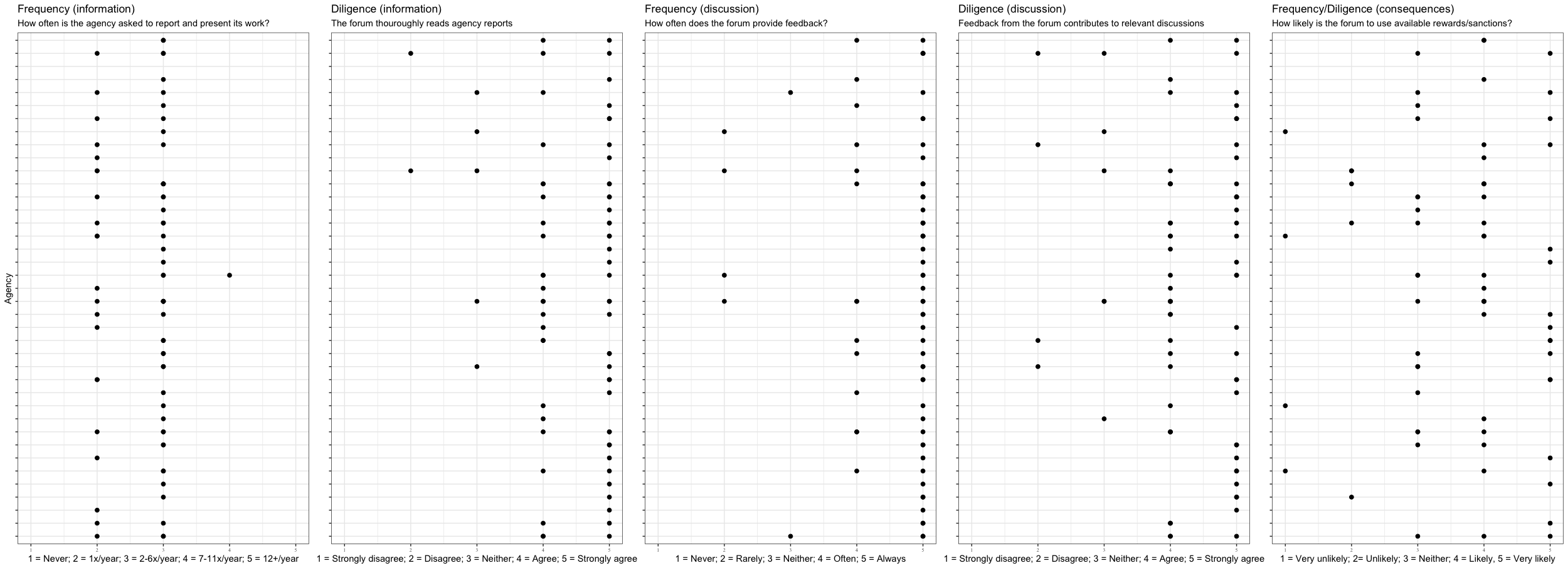


Source: Authors’ survey

Figure 1.6: Within-agency variation – EO


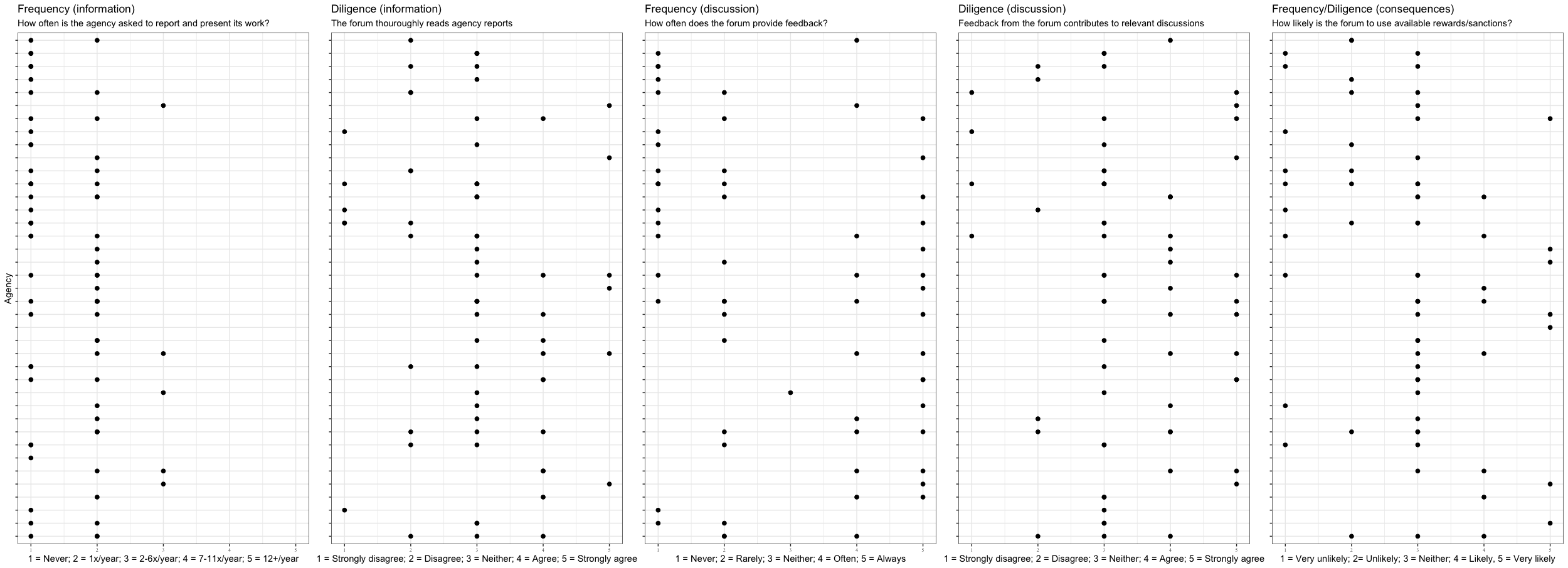


Source: Authors’ survey

*Agency-level Data*

Figures 1.1 – 1.6 show the within agency-variation to responses to the survey questions. We can observe that perceptions about how much account-holding frequency/diligence that an agency is subject to are not completely homogeneous within agencies. In other words, different respondents within the same agency sometimes report that the agency is subject to different levels of account-holding. In addition to the individual level data reported in the article, we are therefore also reporting agency-level data in this appendix for comparison. Informed by the notion that most senior officials are the most involved in accountability processes (see Bovens 2005, p. 203), we assume that they are also the most knowledgeable about these processes. As such, we have selected the highest-level respondent from each agency^[[1]](#footnote-1)^ to represent the agency. Comparing this data (see Figures 1.7 – 1.12) with the individual level data reported in the article (Figures 1 - 6), we see that the results are not substantively different. Consequently, the analysis and the conclusions would not have substantively differed, would we have replaced the individual level data with the agency level data.

Figure 1.7: European Parliament


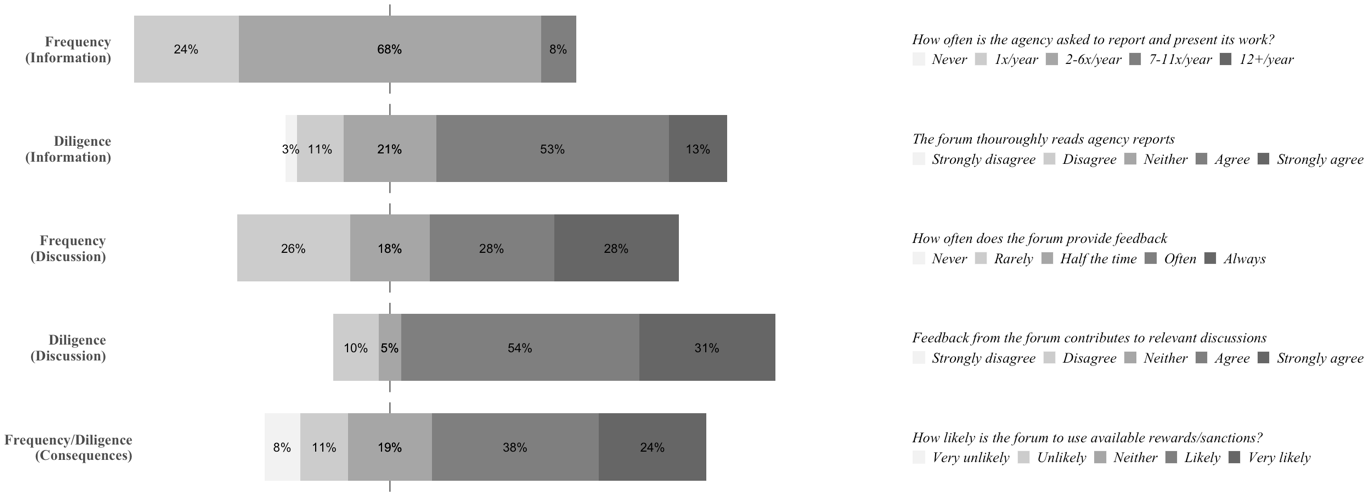


Source: Authors’ survey

Figure 1.8: Council of the European Union


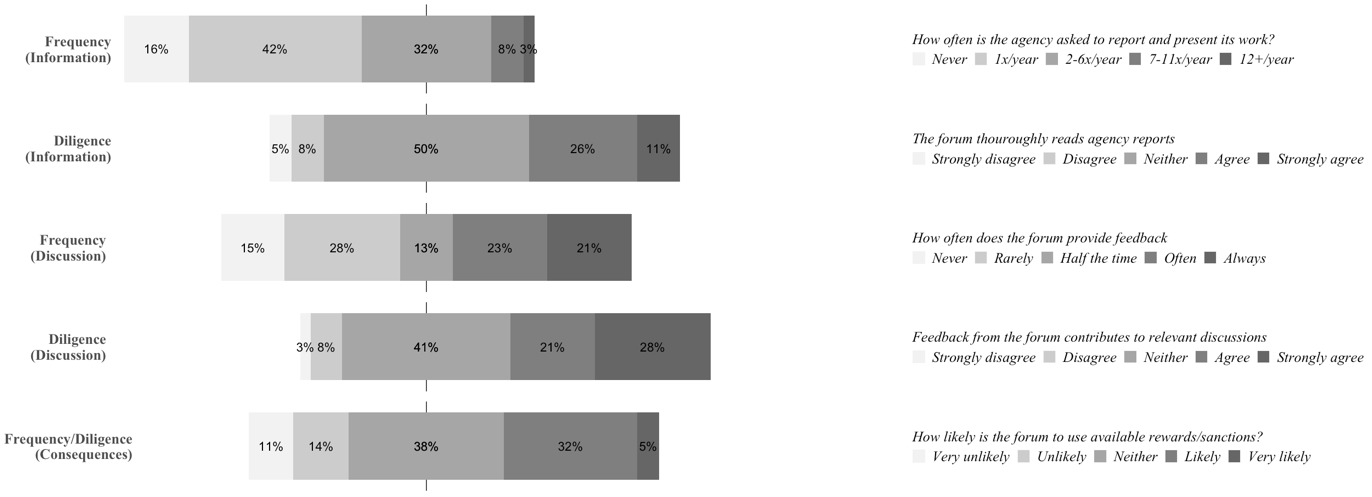


Figure 1.9: European Commission


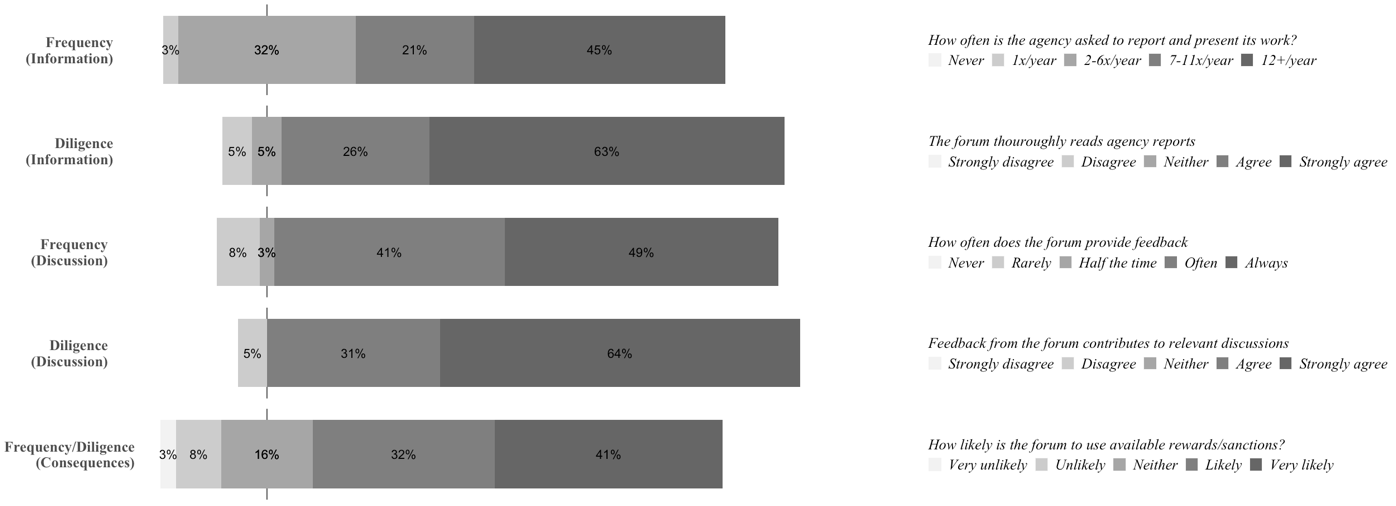


Source: Authors’ survey

Figure 1.10: Management Boards


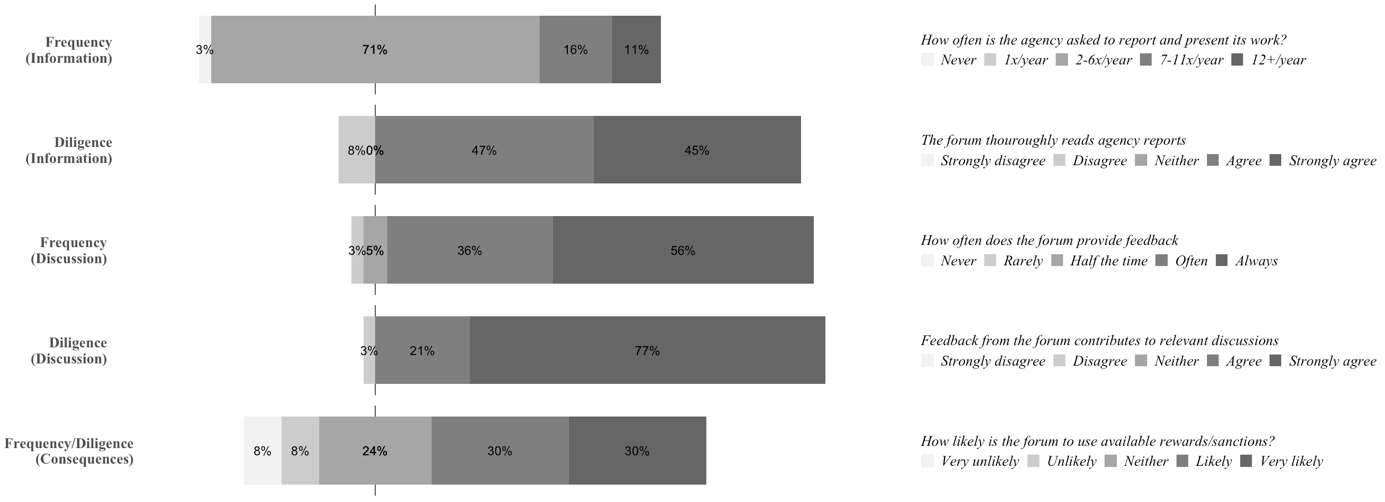


Source: Authors’ survey

Figure 1.11: European Court of Auditors


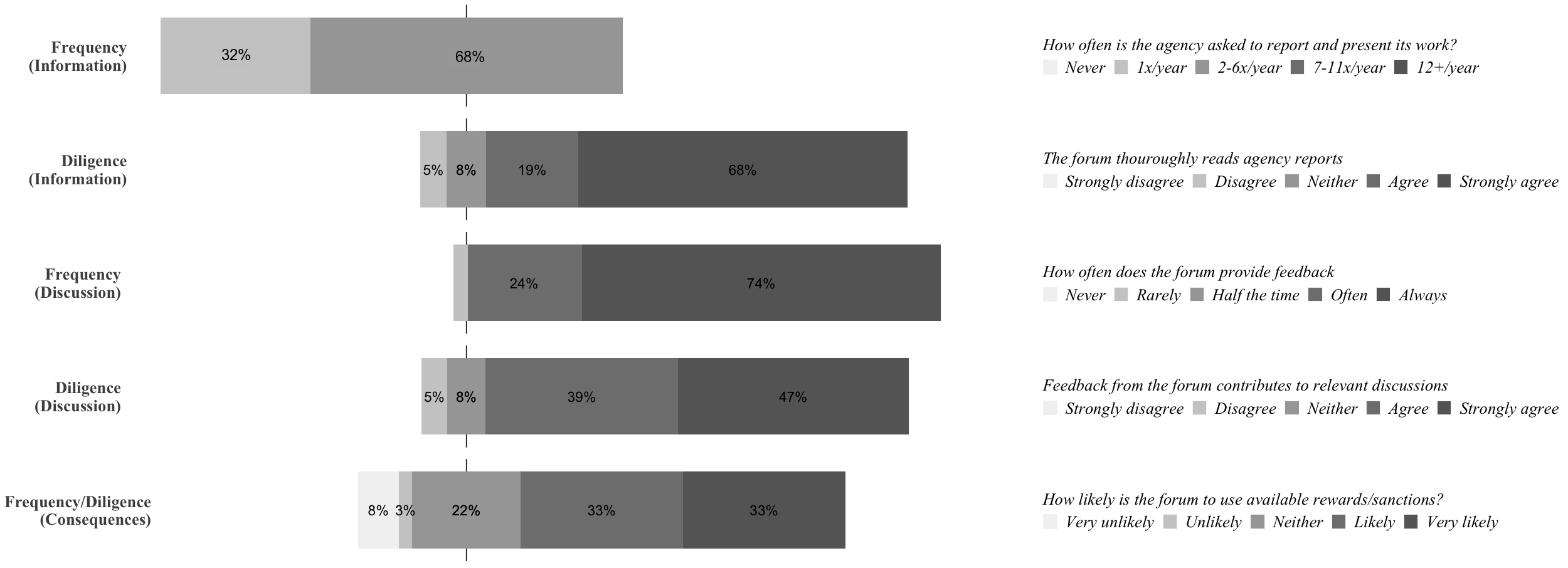


Source: Authors’ survey

Figure 1.12: European Ombudsman


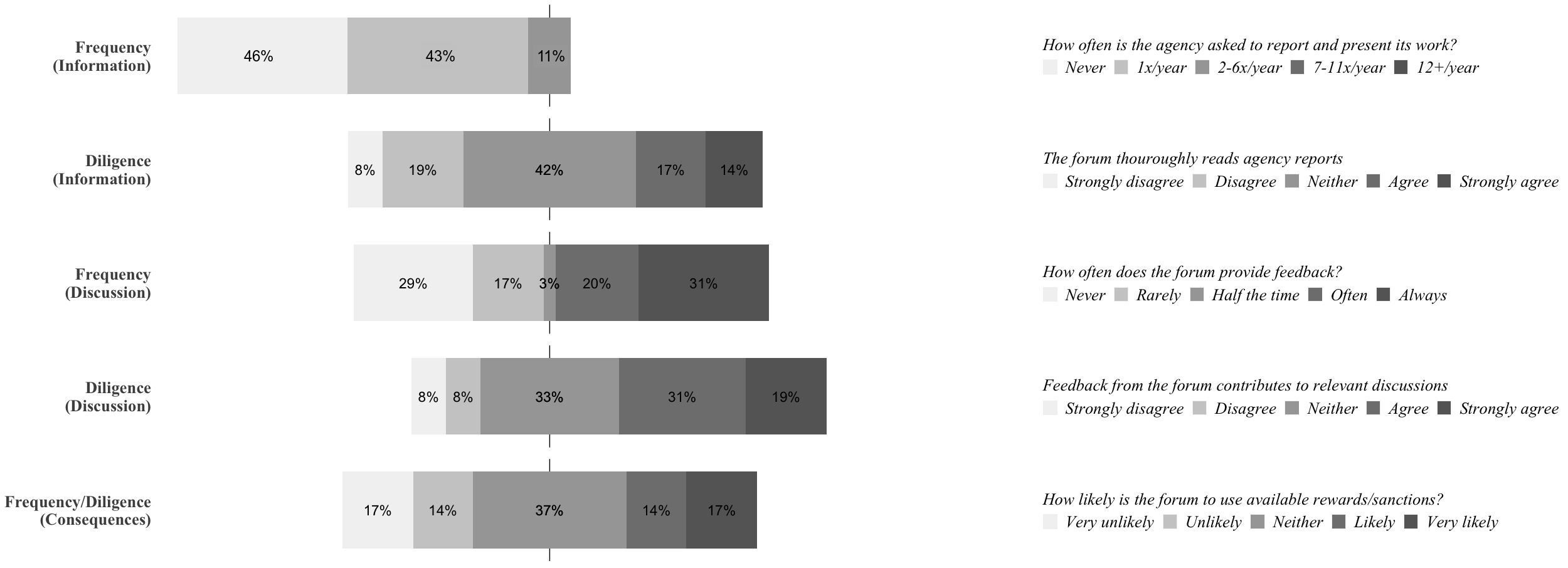


Source: Authors’ survey

**References**

Bovens, M. (2005). Public Accountability. In E. Ferlie, L. E. Lynn Jr., & C. Pollitt (Eds.), *The Oxford handbook of Public Management*. Oxford University Press.

**II: Interview Data**

Table 2.1: Agency Interviews – Organisational positions and agency characteristics

| **Interview #** | **Position** | **Decentralised / Joint Undertaking** | **Regulatory Powers (Soft / Hard)** | **Formal Independence** |
| --- | --- | --- | --- | --- |
| 1 | Other | Joint Undertaking | No | Low |
| 2 | Director + Other | Decentralised Agency | Hard | High |
| 3 | Director | Decentralised Agency | No | Medium |
| 4 | Other | Decentralised Agency | Soft | Medium |
| 5 | Director | Decentralised Agency | Hard | High |
| 6 | Director | Joint Undertaking | No | Medium |
| 7 | Director | Decentralised Agency | No | Medium |
| 8 | Director | Decentralised Agency | Hard | High |
| 9 | Director | Decentralised Agency | No | Medium |
| 10 | Director | Decentralised Agency | Hard | Medium |
| 11 | Deputy Director | Decentralised Agency | No | High |
| 12 | Other | Decentralised Agency | Soft | Low |
| 13 | Other + Other | Decentralised Agency | Soft | Medium |
| 14 | Director + Other | Joint Undertaking | No | Low |
| 15 | Director + Other | Decentralised Agency | Hard | Medium |

Source: Agency #1-15. October 2019 – March 2020. Personal Interviews

In Table 2.1, EU agencies that ‘regulate by information’ (Majone 1997) have been categorized as agencies with ‘soft’ regulatory powers (see Busuioc & Rimkute 2019 for a categorisation of EU regulatory agencies that follows this definition). EU agencies that also have the authority to adopt binding acts (see Busuioc & Jevnaker, 2020, Annex Table 2) have been categorized as agencies with ‘hard’ regulatory powers.

The authors have measured the formal independence of the EU agencies, drawing on Wonka’s and Rittberger’s (2010) independence index, while adapting to relevant critique of said index (Hanretty & Koop 2012). The independence scores range from -1.3923 to 1.8623. In this table, agencies have been categorized as follows: Independence scores < -0.6060 (1^st^ quartile) = Low Independence; -0.6060 (1^st^ quartile) < Scores < 0.5721 (3^rd^ quartile) = Medium Independence; Scores > 0.5721 (3^rd^ quartile) = High Independence.

Table 2.2: Forum Interviewees

| **N** | **Forum** | | |
| --- | --- | --- | --- |
|  | **Council** | **ECA** | **EP** |
| 11 | 1 | 9 | 1 |

Source: Council #1. December 2019. Personal Interview; ECA #1-9. March – October 2019. Personal Interviews; EP #1. October 2019. Personal Interview

**III: Unobtrusive Data**

Table 3.1: Unobtrusive Account-holding Data

| **Agency** | **EP written questions (eighth term) mentioning an agency** | **Council meeting minutes (2015-19) mentioning an agency** | **ECA special reports (2015-19) with an agency as auditee** | **EO opened cases (2015-19) concerning an agency** |
| --- | --- | --- | --- | --- |
| ACER | 22 | 7 | 0 | 0 |
| BBI JU | 2 | 1 | 0 | 0 |
| BEREC OFFICE | 8 | 6 | 0 | 1 |
| CdT | 1 | 0 | 0 | 0 |
| CEDEFOP | 6 | 2 | 0 | 7 |
| CEPOL | 5 | 10 | 0 | 1 |
| CLEAN SKY JU | 4 | 0 | 0 | 1 |
| CPVO | 3 | 0 | 0 | 1 |
| EASA | 65 | 5 | 0 | 6 |
| EASO | 36 | 12 | 1 | 10 |
| EBA | 35 | 13 | 1 | 3 |
| ECDC | 49 | 4 | 1 | 2 |
| ECHA | 68 | 3 | 0 | 6 |
| ECSEL JU | 1 | 0 | 0 | 0 |
| EDA | 8 | 13 | 0 | 2 |
| EEA | 17 | 2 | 1 | 2 |
| EFCA | 8 | 2 | 0 | 0 |
| EFSA | 291 | 47 | 1 | 5 |
| EIGE | 19 | 4 | 0 | 0 |
| EIOPA | 15 | 6 | 1 | 0 |
| EIT | 11 | 3 | 2 | 0 |
| EMA | 72 | 8 | 0 | 13 |
| EMCDDA | 23 | 4 | 0 | 1 |
| EMSA | 19 | 0 | 0 | 0 |
| ENISA | 20 | 5 | 0 | 4 |
| ERA | 79 | 3 | 0 | 0 |
| ESMA | 23 | 9 | 1 | 8 |
| ETF | 13 | 0 | 0 | 0 |
| EU-LISA | 6 | 10 | 0 | 0 |
| EU-OSHA | 16 | 2 | 0 | 1 |
| EUIPO | 15 | 2 | 0 | 1 |
| EUISS | 0 | 1 | 0 | 0 |
| EUROFOUND | 22 | 2 | 0 | 1 |
| EUROJUST | 44 | 33 | 0 | 4 |
| EUROPOL | 272 | 34 | 0 | 9 |
| F4E JU | 1 | 3 | 0 | 1 |
| FCH 2 JU | 0 | 0 | 0 | 1 |
| FRA | 43 | 13 | 0 | 5 |
| FRONTEX | 305 | 38 | 2 | 7 |
| GSA | 2 | 2 | 0 | 0 |
| IMI JU | 6 | 1 | 0 | 0 |
| S2R JU | 3 | 2 | 0 | 0 |
| SatCen | 2 | 2 | 0 | 0 |
| SESAR JU | 12 | 1 | 0 | 0 |
| SRB | 12 | 9 | 1 | 7 |

Table 3.2: EP written questions (eighth parliamentary term, 2014-19)

| **N** | **Written questions** |  |
| --- | --- | --- |
|  | **Number of times that written questions mention agencies** | |
| 30965 | 1684 | |
| Source: https://www.europarl.europa.eu/plenary/en/parliamentary-questions.html [Accessed 2020]. | | |

The search engine for ‘Questions and Union acts’ at the European Parliament’s website was used to search for the number of written questions (addressed to the European Commission) in which agencies were mentioned. In total, Members of the European Parliament asked 30965 written questions to the European Commission during the eighth parliamentary term (Table 3.2). Some written questions mention multiple EU agencies. Consequently, the numbers of written questions that mention at least one agency is somewhat lower than the 1684 times (Table 3.1) that written questions mention agencies.

Table 3.3: Council meeting minutes (2015-19)

| **N** | **Meeting minutes** | |
| --- | --- | --- |
|  | **Agency mentioned** | **No agency mentioned** |
| 322 | 166 | 156 |
| Source: https://www.consilium.europa.eu/en/documents-publications/public-register/council-minutes/ [Accessed 2020]. | | |

While some Council meeting documents are not made public, we were able to retrieve 322 Council meeting documents for the period 2015-19. These documents were loaded into Atlas.ti, and was searched automatically for mentions of EU agencies using an auto-coding function in order to identify in how many meeting documents that individual agencies are mentioned (Table 3.1). 166 of these Council meeting documents (51,6%) were found to mention at least one agency (Table 3.3).

Table 3.4: ECA special reports (2015-19)

| **N** | **Special Reports** | |
| --- | --- | --- |
|  | **Agency mentioned** | **Agency as auditee** |
| 148 | 36 | 7 |
| Source: https://www.eca.europa.eu/en/Pages/PublicationSearch.aspx [Accessed 2020]. | | |

The 148 special reports published by the European Court of Auditors for the period 2015-19 were manually downloaded from their website. These reports were coded by hand. First, it was coded in how many reports that individual agencies are mentioned. 36 reports were found to mention agencies (Table 3.4). Some of these reports mention several agencies. Second, since it was found that many reports mention agencies as sources of information, and not as auditees, it was also coded how often individual agencies are subject to the reports specifically as one of the auditees (Table 3.1). An agency was coded as an auditee of a report if at least one of the following was true: the agency name or acronym is in the title of the audit report; the agency name or acronym is explicitly mentioned in the audit question(s); the agency provides a direct response to the audit findings and recommendations. In 7 reports, agencies were found to be auditees (Table 3.4). In some of these reports, several agencies are auditees.

Table 3.5: EO cases opened (2015-19)

| **N** | **Cases opened** | |
| --- | --- | --- |
|  | **Concerning an agency** | **Not concerning an agency** |
| 929 | 110 | 819 |
| Source: https://www.ombudsman.europa.eu/en/search-inquiries [Accessed 2021]. | | |

The search engine on the European Ombudsman’s website was used to search for the number of times that the Ombudsman opened cases concerned the individual agencies in the period 2015-19 (Table 3.1). A few of these cases concern several agencies. In total, the Ombudsman opened 929 cases during this period, 110 of which concern at least one EU agency (Table 3.5).

**References**

Busuioc, M. & Jevnaker, T. (2020) ‘EU Agencies' Stakeholder Bodies: Vehicles of Enhanced Control, Legitimacy or Bias?’ *European Journal of Public Policy*.

Busuioc, M. & Rimkute, D. (2019) ‘Meeting expectations in the EU regulatory state? Regulatory communications amid conflicting institutional demands’. *Journal of European Public Policy***,** Vol. 22.

Hanretty, C. & Koop, C. (2012) ‘Measuring the formal independence of regulatory agencies’. *Journal of European Public Policy,* Vol. 19**,** pp. 198-216.

Majone, G. (1997) ‘The new European agencies: Regulation by information’. *Journal of European Public Policy,* Vol. 4**,** pp. 262-275.

Wonka, A. & Rittberger, B. (2010) ‘Credibility, Complexity and Uncertainty: Explaining the Institutional Independence of 29 EU Agencies’. *West European Politics,* Vol. 33**,** pp. 730-752.

1. Each agency is represented by its highest-ranking respondent. The selection was made accordingly: 1. Executive Director; 2. Deputy Director; 3. Administrative Director; 4. Operational Director. In the cases where all agency respondents were Operational Directors, the respondent with the least amount of missing data was selected. If the amount of missing data was the same, a respondent was chosen at random. [↑](#footnote-ref-1)
